# Farewell to the Lose–Lose Reality of Policing Plant Imports

**DOI:** 10.1371/journal.pbio.1002438

**Published:** 2016-04-19

**Authors:** Dani Zamir

**Affiliations:** The Institute of Plant Sciences and Genetics, Faculty of Agriculture, The Hebrew University of Jerusalem, Rehovot, Israel

## Abstract

In an age of free international shipments of mail-ordered seeds and plants, more policing will not stop the global migration of hitchhiking pests. The solution is in a preemptive response based on an internationally coordinated genomic deployment of global biodiversity in the largest breeding project since the “Garden of Eden.” This plan will enrich the narrow genetic basis of annual and perennial plants with adaptations to changing environments and resistances to the pests of the future.

## Abstract 2

This Perspective asks us to bid farewell to the failed strategy of policing plant imports and embark on genomics-assisted, biodiversity-based breeding to improve plant resilience to changing environments and to the pests of the future.

*“Plan for what is difficult while it is easy; do what is great while it is small*.*”—*S*un Tzu*, The Art of War

When 182 countries become party to a common cause, it is reason to rejoice. Such an opportunity was provided when the Food and Agriculture Organization of the United Nations (FAO) approved the International Plant Protection Convention (IPPC) on December 6, 1951, with the objective of developing and implementing international phytosanitary standards to reduce the risks associated with the spread of plant pests to agriculture and natural ecosystems [1]. Over the years, the IPPC has been amended to enforce safer trade of plants by preventing the entry and spread of new pests. This led to the establishment of dedicated government agencies, usually associated with the ministries of agriculture, which are responsible for inspecting and policing against the entry of pests. These agencies have grown tremendously over the years because their noble mission is simply to explain to the “want to do good” elected officials who are responsible for the allocation of funds. However, the IPPC is currently implementing a losing defensive strategy, for which a scientific alternative based on a broad view of our interconnected global reality is presented below.

## The Loss to Plant Pests

The international effort to prevent entry, establishment, and spread of plant pests is a rearguard battle. In a letter to the editor of *Plant Pathology*, Brasier [2] gave multiple examples showing that the international plant biosecurity protocols are “outmoded, flawed, institutionalized and too ineffectual.” While live plant imports and the incidence of invasive pests are globally on the rise [3], the IPPC implementing institutions continue to put forward additional forestalling regulations, including heavy penalties for biosecurity breaches. While for international travelers it is clear that imports of agricultural products are strictly prohibited, the internet provides unlimited opportunities to obtain mail-ordered seed by free international shipping. Yesterday, on eBay alone, more than 60,000 seed listings were offered, not to mention live plants, while at the same time the IPPC is calling for further fortifications of its Maginot line.

Furthermore, scientific evidence clearly illustrates that the current regulations are flawed in their basis [2], as shown in the following examples:

1. When estimating the risks of a pathogen, the IPPC emphasis is on those entries that have already caused damage, while the unidentified pathogens, estimated to comprise 90% of the threat, are unknown to science and are ignored [2,4].
2. Policing agencies assume that the potential target hosts for a pathogen will be taxonomically related to those affected in the country of origin. Therefore, testing for a particular pathogen is conducted on a subset of the plant species, while in real life, it has been shown that the host range may be much wider in the new environment.
3. The inspections for phytosanitary certificates are often based on visual evaluations of plant health, while some pathogens may be present but in a form that does not cause symptoms (e.g., spores).
4. Aggressive pathogens identified in a particular country may not be recognized as a risky pest by the international community because of inefficient global communication of such threats.

In view of the dangers posed by the growing world commerce of plants [5–7], current solutions rely on the implementation of much stricter inspections by an increasing number of trained personnel, assisted by the ever-improving molecular tools for the identification of pathogens, combined with extensive use of prolonged postentry quarantine, and finally, high penalties for biosecurity breaches. It is noteworthy that these Maginot defenses are also expected to provide some protection against the use of plant pathogens as agroterrorism weapons [8].

## The Loss to Plant Breeding

Natural biodiversity is the engine that drives improvements in crop productivity and resilience to diseases and environmental stresses [9]. Plant geneticists and breeders are charged with the development and release of new varieties, which provide added value to consumers, producers, and the environment. For mapping and rapid deployment of traits in breeding programs, these solutions often rely on the use of wild species and local varieties as a source of desirable phenotypes and the fruits of genomics [10]. These precious biodiversity resources are crossed to the cultivated varieties, and the traits of interest are incorporated through classical genetic means. In this age of genomic markers, the products are rapidly available for use, and in many cases, the underlying gene or genes are cloned. From a review of the status of gene banks of crop plants, it is clear that collections that were made in the center of origin of a species now serve as valuable resources for thousands of breeders throughout the world. Thus, a resistance gene discovered in a wild species, which is otherwise completely unadapted to modern agriculture, can be found 10 years later in commercial varieties. For example, the whitefly-transmitted tomato yellow leaf curl virus (TYLCV) had spread throughout the temperate regions of the globe starting in the 1990s. At the same time, our lab was developing exotic libraries [9] of wild tomato species, and in 1994, we mapped the*TY-1* resistance gene to chromosome 6 of the *Solanum chilense* library [11] and released the seed and the marker to the Tomato Genetics Resource Center (TGRC) in the University of California, Davis. By the year 2000, most seed companies had tolerant varieties with *TY-1*. In the meantime, the gene was cloned [12], and additional resistance sources were identified, but alas, the TGRC was blocked by new IPPC regulations from sending seed to Israel and to additional countries. Of course, in many cases when breeders use wild genetic resources to improve crop resilience, it is not possible to ascertain if the original seed used as a source of the traits was obtained in accordance with all the phytosanitary regulations. We can safely assume that breeders often cut through the red tape, particularly considering the ease of doing so—i.e., sending the seed in a regular airmail envelope. Thus, when we count the examples of possibly invasive plant pathogens, it is worthwhile to remember that legally and illegally imported plants were the source of numerous novel resistances that protect agriculture and the environment from the damages of pests and pesticides.

To increase their efficiency, the IPPC enforcing organizations are continuously modifying the protocols for seed imports. Consignments of seeds for exports must be examined before they can be sent to ensure that shipments meet the plant health requirements of the importing countries. In most cases, samples of the seeds will be examined in a laboratory to check for the presence of pests and diseases; however, as more and more potential pests are becoming known to the scientific community, the list of tests is expanding such that very few national testing labs are willing to provide a signed official phytosanitary certificate. As a result, it is close to impossible to meet the requirements of the preexport examinations and certification protocols, which effectively blocks legal seed shipment to breeders and geneticists, who are the only authorities that can provide protection in case of entry and spread of a new pest. Thus, the zeal of the IPPC to fulfill its mission is in fact slowing down the progress in incorporating effective and sustainable resistances to plant varieties.

## Breeding Defenses for the Future

The lose–lose scenario of policing seed imports calls for a different strategy to reduce the risks of known and unknown pests through coordinated preparation for the days when the first lines of defense are broken. The culmination of such a collapse is just a matter of time, since we are becoming a global village in so many ways, including in plant resource sharing and pest migration [3,5,6]. The doomsday scenario of the Dutch elm disease was successfully used to demonstrate the dangers of uninvited plant pests. Millions of elm trees in Europe were destroyed by species of the Ascomycete fungus (*Ophiostoma*) that apparently arrived from eastern Asia [2]. The second chapter of the elm disease saga is much more optimistic and provides the basis for this forward-looking proposal. Over the years, it turned out that different accessions of elm showed resistance to the disease, and interspecific hybrids often provided good protection [13]. Thus, if organisms of a narrow genetic basis are challenged with the rich global polymorphism of the pathogen, the only solution is to deploy the full force of the diversity of the attacked organisms.

**Fig 1:**
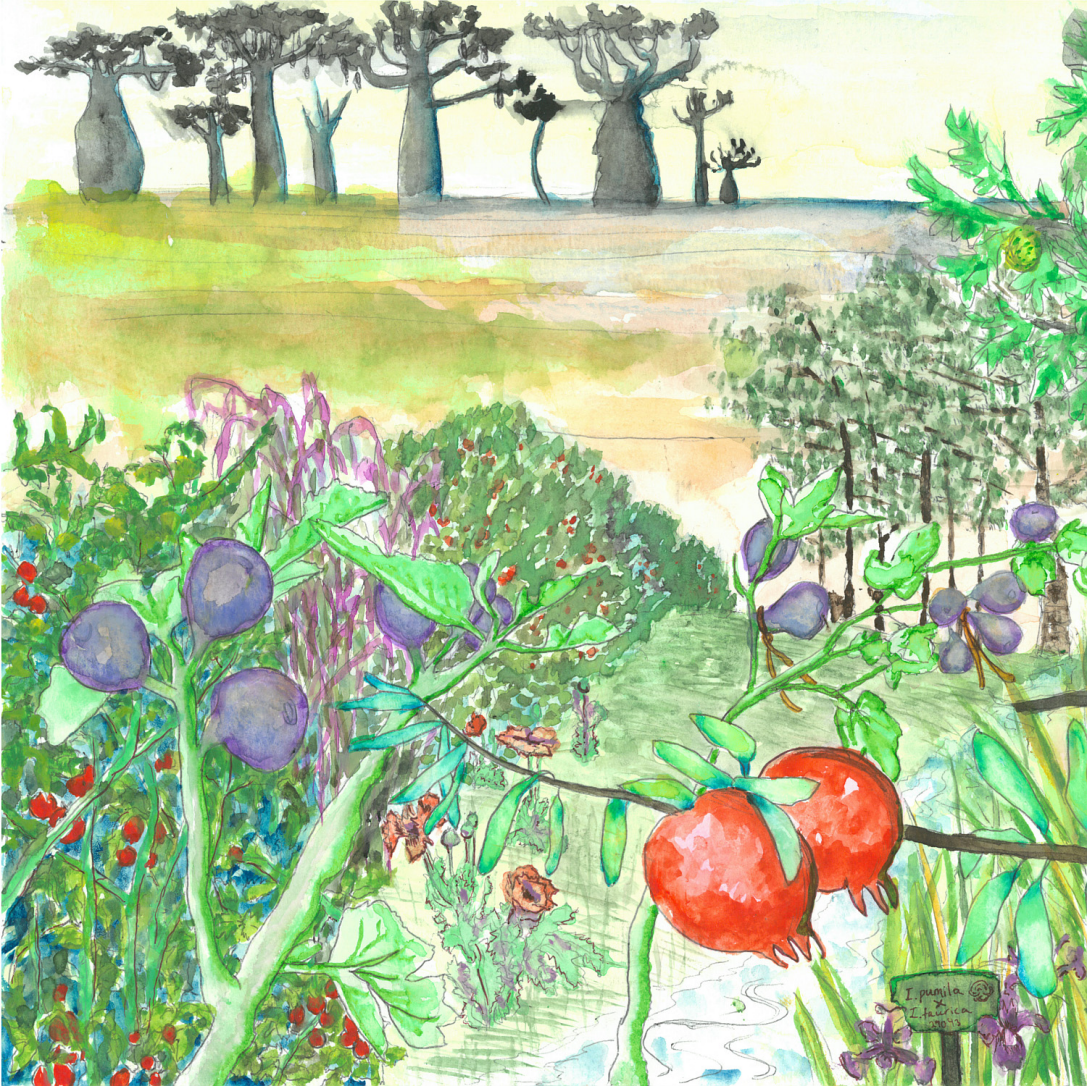
The “Garden of Eden” for plant breeders (by Rachel Meyer).

The proposed conceptual change in our response to future pests is based on maintaining the inclusive international framework of the IPPC and using its organizational strength and resources as a springboard to launch a global preemptive biodiversity-based breeding project (Fig 1). It is important to emphasize that here I develop the scientific rational for the project and evade issues of governance, financing, the identity of the species involved in the hybridization efforts, and the manner of involvement of the private sector. The objective of this commentary is to kick-start a debate that will lead to the evolution of international frameworks that will go beyond the improvement of the resilience of plants to pests. This international breeding effort will involve sites in biodiversity-rich countries that will build the scientific capacity of trained personnel and the appropriate infrastructure to implement a genetic crossing plan involving diverse accessions and wild species of hundreds of annual and perennial plants species. The created plantations and seed of the “mixed breeds” will be shared among the sites and will be challenged with local pests by planting them in different environments and recording their health and development. Other components of the project that will be centrally implemented include the following:

1. **Genomics:** The populations of plants and their pathogens will be sequenced to provide a firm basis of genetic markers that will be used to tag valuable traits and for germplasm identification in a manner that will help in eliminating the barriers to germplasm transfer imposed by the Biological Diversity Convention [8]. According to the recently ratified Nagoya Protocol, the benefits derived from developments made on the basis of biodiversity collected after 1992 must be shared with the country of origin. The complexity of monitoring the origins of the valuable biodiversity leads to a situation in which countries are reluctant to make available their genetic resources. However, in the proposed plan all the germplasms involved in the crosses will carry complete passport data describing their origin as well as complete genome sequence such that single nucleotide polymorphism (SNP) diversity will be an unequivocal means to monitor trait origins and allow for fair and equitable sharing of benefits. However, there are plenty of pre-1992 seed collections that were never used in breeding [14] and could provide a noncontentious starting point.
2. **Informatic:** The project will be linked through a dedicated cloud-based network to provide common software, based on shared vocabulary descriptors, to handle and integrate plant and pest genomes, phenotypes, systematics, and global distribution. This is similar to the Global Health Observatory resources that were established to provide access to over 1,000 indicators on human health priority topics. The Group of Earth Observation’s Biodiversity Observation Network (GEO BON), which develops methods and tools for assessment, analysis, and visualization of global biodiversity information [15], is one example of an ideal partner for the proposed project.
3. **Seed and plant storage:** While it is important to start the hybridizations and create new breeding populations, we aim to keep the seed or plant parts of all generations both for future research purposes and for benefit sharing.
4. **Training and research:** The setting up of a diversity reshuffling by genetic hybridizations depends on the creation of an alliance that will include scientists with a range of expertise who will characterize the genotypes and phenotypes of the heterogeneous populations of plants and their pests. This will be done through partnerships with scientists in academic institutions that will develop new biological concepts, curriculums, and tools based on the data gathered in the project. The objective is not to establish new centers of genomics, informatics, seed storage, or education but rather to partner with strong existing entities interested in participating in the proposed expedition. We are now challenged to find the best route to progress in a proactive and inclusive manner from a defensive position that cannot be policed to the science-based breeding offensive.

The genetic approach for breeding defenses for the pests of the future will generate much broader solutions to related challenges facing our planet. Improvements of the resilience of plants to climate change and adaptation to environmentally friendly agriculture including improved productivity and nutritional value of crops can all be propelled by the biodiversity breeding plan proposed here [9,10,14]. Thus, by bidding farewell to the strategy of policing plant imports and implementing a genomics-assisted biodiversity-breeding strategy, we will be preparing for difficult future challenges while it is still easy to do so.

## Abbreviations

FAO: Food and Agriculture Organization of the United Nations
GEO BON: Group of Earth Observation’s Biodiversity Observation Network
IPPC: International Plant Protection Convention
SNP: single nucleotide polymorphism
TGRC: Tomato Genetics Resource Center
TYLCV: tomato yellow leaf curl virus
